# Validity of Danish register diagnoses of myocardial infarction and stroke against experts in people with screen-detected diabetes

**DOI:** 10.1186/s12889-019-6549-z

**Published:** 2019-02-22

**Authors:** Else-Marie Dalsgaard, Daniel Rinse Witte, Morten Charles, Marit Eika Jørgensen, Torsten Lauritzen, Annelli Sandbæk

**Affiliations:** 1 Department of Public Health, Aarhus University, Bartholins allé 2, DK-8000 Aarhus C, Denmark; 2 Department of Public Health, Aarhus University and Danish Diabetes Academy, Odense, Denmark; 3 Steno Diabetes Center Copenhagen, The Capital Region of Denmark, Copenhagen, Denmark; 4 Department of Public Health, Aarhus University and Steno Diabetes Center, Aarhus, Denmark

**Keywords:** Cardiovascular disease, Predictive value test, Registers, Hospital records, Diabetes mellitus, Type 2

## Abstract

**Background:**

Administrative patient registers are often used to estimate morbidity in epidemiological studies. The validity of register data is thus important. This study aims to assess the positive predictive value of myocardial infarction and stroke registered in the Danish National Patient Register, and to examine the association between cardiovascular risk factors and cardiovascular disease based on register data or validated diagnoses in a well-defined diabetes population.

**Methods:**

We included 1533 individuals found with screen-detected type 2 diabetes in the ADDITION-Denmark study in 2001–2006. All individuals were followed for cardiovascular outcomes until the end of 2014. Hospital discharge codes for myocardial infarction and stroke were identified in the Danish National Patient Register. Hospital medical records and other clinically relevant information were collected and an independent adjudication committee evaluated all possible events. The positive predictive value for myocardial infarction and stroke were calculated as the proportion of cases recorded in the Danish National Patient Register confirmed by the adjudication committee.

**Results:**

The positive predictive value was 75% (95% CI: 64;84) for MI and 70% (95% CI: 54;80) for stroke. The association between cardiovascular risk factors and incident cardiovascular disease did not depend on using register-based or verified diagnoses. However, a tendency was seen towards stronger associations when using verified diagnoses.

**Conclusions:**

Our results show that studies using only register-based diagnoses are likely to misclassify cardiovascular outcomes. Moreover, the results suggest that the magnitude of associations between cardiovascular risk factors and cardiovascular outcomes may be underestimated when using register-based diagnoses.

## Background

Administrative patient registers are often used to evaluate health planning activities and to assess the occurrence of disease events and morbidity in epidemiological studies and trials. The validity of register data is thus very important.

Hospital discharge diagnoses in Denmark have been registered in the Danish National Patient Register (DNPR) since 1997 and classified according to the International Classification of Disease coding system (ICD-8 between 1997 and 1993 and ICD-10 since 1994) [1]. The DNPR are found to have good overall coverage and holds data for 99.4% of all Danish somatic hospital discharges [2, 3]. There is no gold standard for evaluating diagnosis, which is why only the likelihood of a correct diagnosis can be assessed.

Previous studies of the positive predictive value (PPV) of cardiovascular diseases in the DNPR have generally found fair agreement between the registered codes and hospital records [4–13]. A recent Danish study evaluating more than 2000 discharge codes for cardiovascular diagnoses in 2010–2012 found that the PPV of cardiovascular disease based on hospital records as the reference standard varied according to diagnosis; PPV for first time myocarditis:64% and for first time myocardial infarction (MI):97% [12]. Other studies have found PPVs in the range 81–100% [4, 5, 7, 9]. Previous studies of the validity of stroke in the DNPR have shown PPVs in the range 92–97% [6, 11, 13]. The methods for validating the diagnoses varied in populations, settings, reference standards and validation procedures, which may explain the differences seen in reported PPVs. In this study, we wanted to validate register-based event codes against events validated by experienced clinicians. Besides discharge summaries and medical records, we included electrocardiographs, laboratory tests, post-mortems (autopsies) and death certifications in the determination of the reference diagnosis.

The aim of the study was to assess the PPV of register-based MI and stroke events compared with diagnoses validated by adjudication experts in a screen-detected type 2 diabetes population. Furthermore, we aimed to examine the potential impact of using register-based events compared to validated diagnoses on the association between cardiovascular risk factors and cardiovascular events.

## Methods

### Population

The study population comprised 1533 individuals identified with type 2 diabetes by screening in the Danish part of the ADDITION study (Anglo-Danish-Dutch Study of intensive Treatment in People with Screen-Detected Diabetes in Primary Care) in 2001–2006. All individuals were aged 40–69 years at inclusion in the study. The overall aim of the ADDITION study was to examine whether early detection and early treatment onset could reduce cardiovascular events among people with screen-detected type 2 diabetes in a randomised controlled trial. The primary outcome of the study was a composite of cardiovascular events, including cardiovascular morbidity and cardiovascular mortality. The ADDITION study is described in detail elsewhere [14, 15]. The study population had a mean diabetes duration of 11.6 years.

### Data

Data from the DNPR were linked at the individual level through the personal registration number, which is a unique identification number provided to all Danish citizens. The DNPR holds information on all admissions since 1977 to Danish somatic hospitals and outpatient clinics, including discharge dates and discharge diagnosis. The DNPR was searched for cardiovascular disease as primary diagnosis according to the International Classification of Disease, 10th revision. We identified myocardial infarction (ICD-10 codes: I21–24) and stroke (ICD-10 codes: I61–65) from the date of inclusion in the ADDITION study until the end of 2014. For each event, medically trained staff collected and organised all relevant clinical information. This included hospital medical records, hospital discharge summaries, electrocardiographs, laboratory results, death certifications, post mortems, autopsies, descriptions of images, e.g. X-rays, MRI and CT scanning, and notes from general practitioners.

We excluded individuals who had experienced MI or stroke during the last five years before the inclusion in the ADDITION study, and we included only first presentation of the relevant diagnosis code in the study period.

### Adjudication

An independent adjudication committee consisting of four experienced clinicians (three in cardiology and one in diabetology) evaluated each potential event. The above-mentioned material was randomly sent to two members of the committee, and each member evaluated the potential events independently. The evaluation followed a predefined adjudication manual (Table 1), and a uniform adjudication form was completed for each potential event. Furthermore, if members of the committee had insufficient information to determine a case, they could apply for supplementary material to help make a correct decision. In case of disagreement between two evaluators, the material was sent to all four members of the committee for assessment. Consensus was then obtained in a subsequent meeting between all members of the adjudication committee.

**Table 1:** Guidelines for adjudication of potential events of MI and stroke in the ADDITION-study

MYOCARDIAL INFARCTION (MI)	
Fatal	
Death from a new (within 30 days) acute myocardial infarction (MI)	
Confirmed in hospital by appropriate biochemistry, ECG or imaging test * or*	
Confirmed by autopsy showing a recent MI or recent occluding coronary thrombus, whether or not the patient was in hospital	
Non-fatal	
The term MI should be used when there is evidence of myocardial necrosis in a clinical setting consistent with myocardial ischemia. Under these conditions, any one of the following criteria meets the diagnosis of MI:	
• Detection of rise/fall of cardiac biomarkers (preferably troponin) with at least one value above the 99th percentile of the upper reference limit (URL) together with evidence of myocardial ischemia with at least one of the following:	
• Symptoms of ischemia	
• ECG changes indicative of new ischemia (new ST-, T- changes or new left bundle branch block (LBBB))	
• Development of pathological Q waves in the ECG	
• Imaging evidence of new loss of viable myocardium or new regional wall motion abnormality.	
STROKE	
Fatal	
Death occurring within 30 days from the onset of symptoms suspected to be due to a cerebrovascular event, including athero/thrombotic infarction, embolism or haemorrhage, assuming no other more relevant intervening event. In the absence of other obvious causes for the sudden onset of neurological signs and symptoms, the endpoint committee should presume a vascular cause. Death due to subarachnoid or subdural haemorrhage should be included in this category. A stroke may also be defined by autopsy findings showing a recent cerebrovascular event, including athero/thrombotic infarction, embolism or haemorrhage, whether or not the patient was in hospital	
Non-fatal	
The diagnosis of stroke requires evidence of a neurological deficit, usually localised, lasting 24 h or more, usually confirmed by diagnostic testing (e.g. CT scan). The clinical characteristics of stroke include sudden onset of a neurological deficit, typically manifested as:	
• Depression of state of consciousness	
• Disturbance of vision	
• Paresis of paralysis of one or more extremities	
• Sensory impairment	
• Speech impairment	
• Central cranial nerve dysfunction	
• Memory defect	
• Ataxia	
• Movement disorder	
Confirmed diagnoses of stroke will be categorised into the following:	
1. *Definite ischemic stroke:* verified by CT or MRI scanning carried out within 2 weeks after the stroke (focal neurological deficit of more than 24 h of duration) or by autopsy.	
2. *Definite hemorrhagic stroke:* primary intracerabral, subarachnoidal or secondary to ischemic stroke verified by CT or MRI scanning within 2 weeks after the stroke (focal neurological deficit of more than 24 h of duration) or by autopsy or lumbar puncture.	
3. *Not classifiable*	

*MI* myocardial infarction, *ECG* electrocardiogram, *CT* computer tomography, *MRI* magnetic resonance imaging**,** *URL* upper reference limit, *LBBB* left bundle branch block

### Cardiovascular risk factors

We obtained baseline information on selected cardiovascular risk factors: HbA_1c_, cholesterol, blood pressure, BMI, and smoking status. This information was collected at the inclusion in the ADDITION study from clinical examinations and self-administrated questionnaires.

### Statistics

The PPV of the diagnoses of MI and stroke was assessed as the proportion of the diagnoses identified in the register that were confirmed by the adjudication committee. In other words, the numerator was cases confirmed by the adjudication committee and the denominator was the number of potential cases identified in the DNPR. The results were stratified by calendar time and by sex. The diagnosis of stroke was further subdivided into hemorrhagic, ischaemic and non-specified stroke. The association between cardiovascular risk factors at baseline and register-based or verified diagnosis of CVD, respectively, was examined by Cox proportional Hazards. CVD was defined as a composite of MI and stroke. Analyses were adjusted for study randomisation while accounting for clustering by general practitioner. Results were displayed graphically in forest plots. Risk estimates were assessed with 95% confidence intervals (CI). All analyses were performed in Stata 14.

## Results

A total of 1533 individual identified with screen-detected type 2 diabetes were followed for diagnosis of MI and stroke; mean follow-up: 11.6 years. Of 69 identified first-events of MI in the DNPR the adjudication committee confirmed the diagnoses of 52 (75%). (Table 2). Among the non-confirmed cases, two individuals had experienced coronary artery bypass graft surgery, two had had percutaneous coronary interventions, and one had died from cardiac arrhythmic complications on the date of the MI diagnosis in the DNPR. We identified 17 women with MI diagnosis in the DNPR; 88% were confirmed by the adjudication committee. We identified 52 men with MI; 71% were confirmed.

**Table 2:** PPV of CVD diagnosis in the Danish National Patient Register verified by the adjudication committee

	MI *N* = 1533	Stroke *N* = 1533
Previous CVD event, *n*	53	40
Eligible for assessment, *n*	1479	1490
Diagnosed in DNPR, *n* (% of eligible)	70 (4.7%)	49 (3.1%)
Hospital record available, *n* (% of eligible)	69 (4.7%)	46 (3.1%)
Confirmed by adjudication committee, *n* (% of eligible)	52 (3.5%)	32 (2.1%)
PPV, % (95% CI)	75% (64; 84%)	70% (54; 80%)

*DNPR* Danish National Patient Register, *MI* myocardial infarction, *PPV* positive predictive value

Among the 46 cases identified with first event of stroke in the DNPR the adjudication committee confirmed 32 cases (70%) (Table 2). Confirmation rates for stroke subgroups are presented in Table 3. We identified 18 women with a diagnosis of stroke in the DNPR; 61% were confirmed by the adjudication committee. We identified 28 men with a diagnosis of stroke in DNPR; 75% were confirmed. We tested for the effect of calendar time and found no significant difference in PPVs over time for either MI or stroke.

**Table 3:** Diagnosis of stroke in the Danish National Patient Register by sub-diagnosis and confirmation rates

ICD-10 codes		*N* = 46	Verified, *n* (%)
I60-I62	Haemorrhage	5	3 (60%) 1 (20%) Cerebrovascular stroke death
I63	Cerebral infarction	23	18 (78%) 1 (4%) non-classified
I64	Stroke, non-classified	18	9 (50%)

*ICD-10* International Classification of Disease, 10th Revision; *DNPR* Danish National Patient Register

### Cardiovascular risk factors

**Fig. 1:**
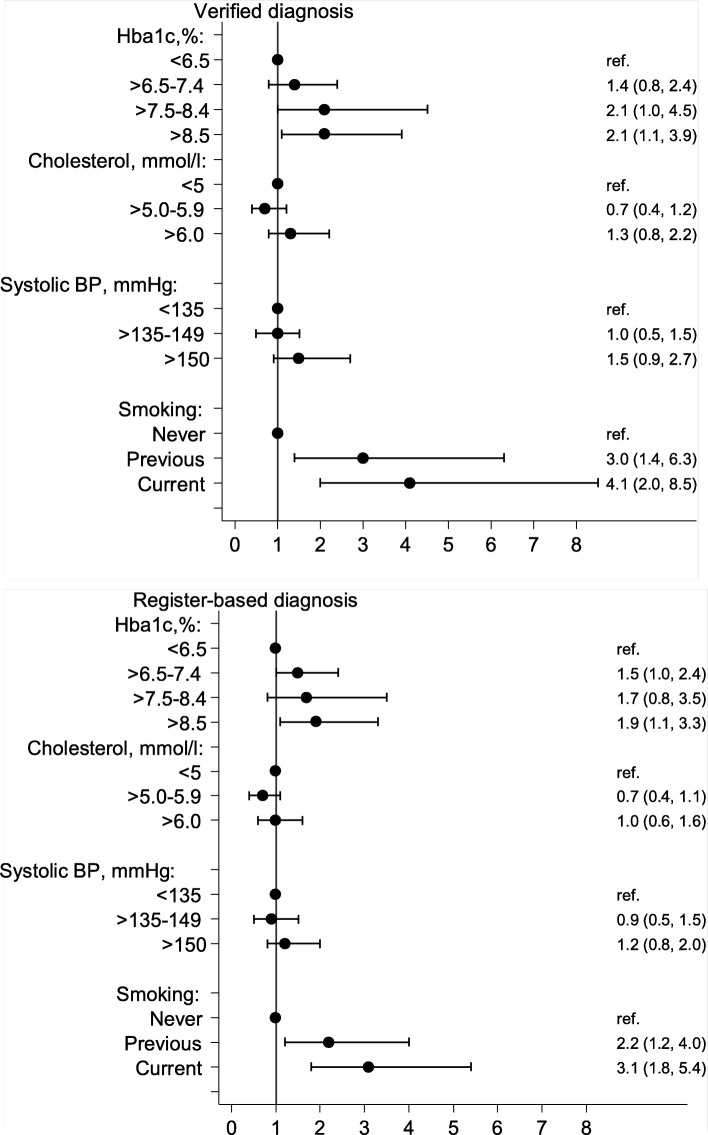
The association between cardiovascular risk factors and CVD registered in the Danish National Patient Register or verified by adjudication committee, HR (95% CI). CVD: cardiovascular disease defined as diagnosis of MI or stroke; DNPR: Danish National Patient Register; Hba_1c:_ glycated haemoglobin; BP: blood pressure; HR: hazard ratio; ref.: reference group (HR = 1)

We examined the association between selected cardiovascular risk factors (HbA_1c_, total cholesterol, blood pressure and smoking status) and the incidence of CVD (diagnosis of either MI or stroke) identified in the DNPR or verified by the adjudication committee, respectively (Fig. 1). We found no statistically significant difference between the two approaches, although we saw a tendency towards a stronger association between high HbA_1c_ or smoking and CVD based on the verified diagnosis than CVD based on the register-based diagnosis.

## Discussion

In this study, we examined how well the national registers in Denmark capture diagnoses of MI and stroke, compared to a diagnosis verified by an adjudication committee consisting of experienced clinicians. We found that using a diagnosis based on registers overestimates the incidence rate of both MI and stroke in a population with screen-detected type 2 diabetes. The adjudication committee verified only three out of four of the register-based diagnoses of MI and stroke. Furthermore, we examined the associations between cardiovascular risk factors at baseline and incident CVD during follow-up (mean follow-up: 11.6 years). We found that the presence of associations did not generally depend on whether the diagnoses were based on registers or clinical verification. However, an indication of a stronger association between risk factors and CVD was observed when using verified CVD events, especially for HbA_1c_ and smoking. This finding indicates that although epidemiological studies using register-based diagnoses identify a larger number of events, the fraction that cannot be validated introduces heterogeneity and noise that exceeds any potential gain in statistical power from the larger number. This may thus lead to considerable underestimation of the strength of the association between risk factors and CVD occurrence, despite the larger number of recorded events.

A strength of this study was that all cases were evaluated based on a broad range of accessible medical information. In addition, medically trained staff carefully collected and organised this material. Moreover, data was achieved for 96% of the potential cases as only four cases were excluded because of missing data or no available medical record.

An independent adjudication committee consisting of trained and experienced clinicians evaluated all potential events. In case of disagreement between two independent evaluations by clinicians, all four members of the committee evaluated the specific case and consensus was obtained in a consecutive meeting. Consequently, we consider the adjudication of cases in our study to be accurate and correct and similar to the standards used in most randomised controlled clinical trials.

We considered only the first presentations of diagnosis codes of MI or stroke in the NDPR during the study period. This was done to avoid misclassification as the risk of a previous diagnosis in the register might influence the future discharge diagnosis.

A limitation of our study was the relatively small sample size. Although we followed a population of 1533 persons with screen-detected type 2 diabetes during a mean follow-up period of 11.6 years, we identified a limited number of events and thereby had a low incidence rate for both MI and stroke. Therefore, the study had limited power to evaluate the PPVs stratified on several variables, e.g. gender, calendar year, and hospital type.

We collected events from all types of hospitals located in three out of the five Danish regions distributed over the entire country. Consequently, the results can be generalized to individuals with type 2 diabetes in Denmark. However, our results should be regarded as country-specific as they are based on Danish nationwide registers and thus cannot be generalised to other countries.

We found considerably lower PPVs in this study compared to other studies both for MI (PPV: 81–100%) [4, 5, 7, 9] and for stroke (PPV 80–97%) [6, 11]. The differences in confirmation rate might partly be explained by the way the cases were evaluated. Sundboll [12] found a PPV of 97% (91–99%) for MI. One single reviewer evaluated whether the diagnosis in the hospital discharge summary confirmed the ICD-10 code in the DNPR. The medical hospital record was evaluated only if the assessment could not be determined through the discharge summary alone. In case of doubt, a second evaluator was involved in the actual case. A similar approach was used by Thygesen [9], who found a PPV of 98.0% (89.5–99.7%). Medical records were only searched if the discharge summary did not state the diagnosis or if the discharge summary was missing. A single evaluator reviewed the cases, and a second evaluator was only involved in case of doubt. Joensen [5] found a PPV of 81.9% (79.5–84.2%) based on a review of medical records by one single reviewer, whereas Coloma^7^used computer software to verify cases based on medical records and found a PPV of 100% (97.5–100%) for MI.

Krarup [11] found a PPV of 80.5% (73.6–86.3%) for stroke and evaluated cases based on medical records, hospital discharge summaries, diagnostic imaging, autopsy reports and angiography reports. Like in our study, two independent reviewers evaluated all potential cases. Wildenschild [10] found a PPV of 79% (62–88%) for stroke. The diagnosis of stroke was based on a review of medical records and diagnostic imaging. One single reviewer evaluated each case. In case of uncertainty, two consultants in neurology evaluated the case and obtained consensus. Johnsen [13] evaluated cases based on medical records and all available material assessed by one single reviewer and found a PPV of 79.3% (74.9–83.3%) for stroke.

The discrepancy in confirmation rates between our study and others may be explained by the more strict evaluation of cases in our study than in other studies. We considered more available medical information, the included cases were evaluated by two independent clinicians, and consensus was reached in case of doubt. Furthermore, as CVD event was the main outcome measure in the ADDITION study, we strived to determine if each case concerned a CVD event or not. If any doubt remained at the consensus, the case was categorised as ‘not an event’. In summary, only cases in which the diagnosis could be confirmed by 100% were regarded as CVD events.

It is important to consider study setting when comparing PPVs. Joensen [5] found the PPV for MI to be higher (92.4%) for patients discharged from a hospital ward compared to other types of discharges (81.9%). Sundboll [12] identified the potential events of MI in the DNPR based on data from three hospitals in the Central Denmark Region and identified higher PPVs than we did. In our study, we considered all potential events of MI and stroke regardless of setting. This might add to the explanation of our lower confirmation rates.

Sundboll [12] limited the evaluation to the period of 2010–12 and suggested that the PPV increased over time, most likely because of the implementation of clearer guidelines, more specific definitions of disease, and raising awareness of correct coding of entries in registers. Although we examined CVD events over a much longer time span, we were not able to confirm a time trend in our study. This might be caused by our limited sample size. However, epidemiological studies using register-based data as proxies for morbidity will often require information on the diagnoses for a longer follow-up period.

It is important to underline that there is no gold standard for evaluation of diagnosis of CVD and studies can only estimate the likelihood of agreement. Our study and other studies report PPVs as outcome. Based on the available data, it was not possible to estimate the sensitivity and specificity of the register-based diagnoses in this population. Furthermore, we had not the possibility to evaluate if a possible event was missing in the register. Furthermore, in most studies, the incidence of CVD in the population was not available because of the sampling procedure in the study. The PPV varies in different populations because it is closely linked to disease incidence. In our study, we found a relatively low incidence of both MI and stroke, which resulted in the relative low PPVs for both diseases. The age of the population also influences the PPV as incidence rates tend to increase with age. Krarup [11] found higher PPVs for stroke in a population with a mean age of 73 years after two years follow-up than we found after 11 years follow-up in a much younger population. In addition, Krarup stated that comorbidity could make the diagnostic procedure more difficult [11]. This issue must also be considered in our study with a population of people with type 2 diabetes and a mean diabetes duration of 11.6 years. Hence, our study participants have high risk of comorbidity, which might complicate the assessment of events.We found that the presence of associations between cardiovascular risk factors and diagnosis did not differ when using register-based data or a clinically verified diagnosis. However, we saw a tendency towards a stronger association between cardiovascular risk factors and cardiovascular disease when using the verified diagnosis of CVD compared to using register-based diagnosis. In other words, a more reliable diagnosis showed stronger association with the risk factors (data not shown).

The great advantage of record linkage in epidemiological studies is the ability to carry out large-scale event tracing at low cost and high speed. This exceptional asset enables follow-up of very large cohorts or even the country’s entire population, a scale that would otherwise be prohibitively expensive. However, our study indicates that in smaller settings such as clinical trials, the additional investment in clinical event ascertainment may be justified by the added statistical power it yields through better precision.

## Conclusions

This study aimed to assess the PPVs of MI and stroke recorded in the DNPR and compare these with clinically validated diagnoses. We found that studies using diagnosis based exclusively on registers were likely to misclassify cardiovascular outcomes to some degree. Moreover, the results suggest that using register-based diagnoses in epidemiological studies may lead to underestimation of the strength of association between cardiovascular risk factors and cardiovascular disease.

## Abbreviations

ADDITION: Anglo-Danish-Dutch Study of intensive Treatment in People with Screen-Detected Diabetes in Primary Care
CVD: Cardiovascular disease
DNPR: Danish National Patient Register
ICD: International Classification of Disease coding system
MI: Myocardial Infarction
PPV: Positive Predictive Value
